# LLMs achieve adult human performance on higher-order theory of mind tasks

**DOI:** 10.3389/fnhum.2025.1633272

**Published:** 2026-01-02

**Authors:** Winnie Street, John Oliver Siy, Geoff Keeling, Adrien Baranes, Benjamin Barnett, Michael McKibben, Tatenda Kanyere, Alison Lentz, Blaise Agüera y Arcas, Robin I. M. Dunbar

**Affiliations:** 1 Google, London, United Kingdom; 2 Google DeepMind, London, United Kingdom; 3 Applied Physics Lab, Johns Hopkins University, Baltimore, MD, United States; 4 Independent Researcher, London, United Kingdom; 5 Department of Experimental Psychology, University of Oxford, Oxford, United Kingdom

**Keywords:** large language models, theory of mind, AI, social cognition, mentalizing, social AI

## Abstract

This paper examines the extent to which large language models (LLMs) are able to perform tasks which require higher-order theory of mind (ToM)—the human ability to reason about multiple mental and emotional states in a recursive manner (e.g., I *think* that you *believe* that she *knows*). This paper builds on prior work by introducing a handwritten test suite—Multi-Order Theory of Mind Q&A—and using it to compare the performance of five LLMs of varying sizes and training paradigms to a newly gathered adult human benchmark. We find that GPT-4 and Flan-PaLM reach adult-level and near adult-level performance on our ToM tasks overall, and that GPT-4 exceeds adult performance on 6th order inferences. Our results suggest that there is an interplay between model size and finetuning for higher-order ToM performance, and that the linguistic abilities of large models may support more complex ToM inferences. Given the important role that higher-order ToM plays in group social interaction and relationships, these findings have significant implications for the development of a broad range of social, educational and assistive LLM applications.

## 1 Introduction

Theory of Mind (ToM) is the ability to infer and reason about the mental states of oneself and others (Premack and Woodruff, 1978; Wimmer and Perner, 1983; Wellman et al., 2001). ToM is at the core of human social intelligence, facilitating meaningful communication, enabling empathy, and allowing us to explain, predict and influence one anothers' behaviours in a wide range of cooperative and competitive scenarios (Humphrey, 1976; Wellman and Bartsch, 1988; Hooker et al., 2008). ToM is so crucial to human social life, that its deficiencies, which often afflict those with psychiatric disorders (including autism and schizophrenia) or suffering from alcohol abuse, are often associated with poorer interpersonal relationships and compromised quality of life (Harrington et al., 2005; van Neerven et al., 2021; Le Berre, 2019; Mao et al., 2023; Lei and Ventola, 2018).

A question that has begun to concern researchers of large language models (LLMs; Brown et al., 2020; Bommasani et al., 2021; Zhao et al., 2023) is whether or not LLMs possess ToM. LLMs being able to infer the mental and emotional states of people could have wide-ranging implications for user-facing LLM applications. In the first instance, LLM ToM might result in more appropriate, personalised responses to queries and explanations that are tailored to users' needs and level of understanding (Street, 2024). LLM ToM is also likely to be a foundational component for the development of sophisticated social AI agents. People are already interacting with social AI agents in the form of friends, personal tutors and even romantic partners. But we might also imagine such social agents having transformative potential in social skills training for populations with diminished social abilities due to neurological or psychological conditions, or those with diminished access to social interaction.

LLMs have been shown to perform well on some human psychological tests for ToM (Kosinski, 2024; Bubeck et al., 2023; Shapira et al., 2024; Strachan et al., 2024). For example, (Kosinski 2024) argued for spontaneous ToM emergence in LLMs based on GPT-4's success on a suite of tasks inspired by the classic Sally-Anne task^1^ and (Strachan et al. 2024) found that GPT-4 performed at or above human level on ToM tasks including false belief and misdirection. However, the robustness of these findings is disputed and the subject of ongoing research. (Ullman 2023) challenged (Kosinski 2024) claim, demonstrating decreased performance with minor task perturbations and further experiments involving benchmark suites like BigToM (Gandhi et al., 2024) and SocialIQa (Sap et al., 2022) have yielded mixed results. As (Hu et al. 2025) point out, one of the reasons for these disparate results is that there is no consensus within the research community about whether LLM ToM can be established by LLMs matching human *behaviour*, or whether they should also match human *computations*. We will return to this issue in the discussion, but for now suggest that while there remain significant gaps in our understanding of the computational processes underlying LLM outputs, performance on human ToM tasks provides an important starting point for investigating LLM behavioural capabilities and the potential cognitive similarities between LLMs and humans.

Most of the literature on LLM ToM has focused on ToM at the 2nd-order of intentionality (Sap et al., 2022; Kosinski, 2024; Gandhi et al., 2024; Shapira et al., 2024), where the “order of intentionality” is the number of mental states involved in a ToM reasoning process (i.e., a 2nd-order statement“I *think* you *believe*”). The capacity to make second-order intentional inferences is normally achieved by the age of 4 or 5 (Westby and Robinson, 2014). However, adults regularly use *higher-order* ToM, where multiple mental states are considered at once in a nested or recursive manner, and several studies have shown that most normal adults can reach at least 5th-order inferences (e.g., a 5th-order statement would be “I *believe* that you *think* that I *imagine* that you *want* me to *believe*”) (Kinderman et al., 1998; Stiller and Dunbar, 2007; Oesch and Dunbar, 2017).^2^ Given that LLMs are increasingly leveraged for multi-party social interaction contexts with adult humans (Wang et al., 2024; Park et al., 2023), they are likely to need higher-order ToM capabilities to behave appropriately and effectively. A range of evidence suggests that higher-order ToM is important for complex human social interactions. People who have mastery of higher-orders of ToM inferences tend to have more people in their close social networks (Lewis et al., 2011; Stiller and Dunbar, 2007; Launay et al., 2015) and greater overall social competence (Liddle and Nettle, 2006). Greater capacity for ToM up to 5th-order intentionality correlates with mastery of recursive syntax (Oesch and Dunbar, 2017) and perceived humour in jokes is positively correlated with the number of mental states involved (up to 5th-order ToM) (Dunbar et al., 2016). Agent modelling studies have also associated higher-order ToM with improved cooperation (Ridinger and McBride, 2017) and negotiation (De Weerd et al., 2017, 2022) skills. The 5th-order limit on human ToM ability appears to be constrained by cognitive processing power: individual differences in higher-order ToM ability correlate with neocortex size (Lewis et al., 2011) and higher-order ToM inferences recruit disproportionately more neural response than lower-order (Lewis et al., 2017). Higher-order ToM competency also varies within the population, including by gender (Hyde and Linn, 1988; Stiller and Dunbar, 2007), and is not deployed reliably across all social contexts (Keysar et al., 2003).

**Figure 1:**
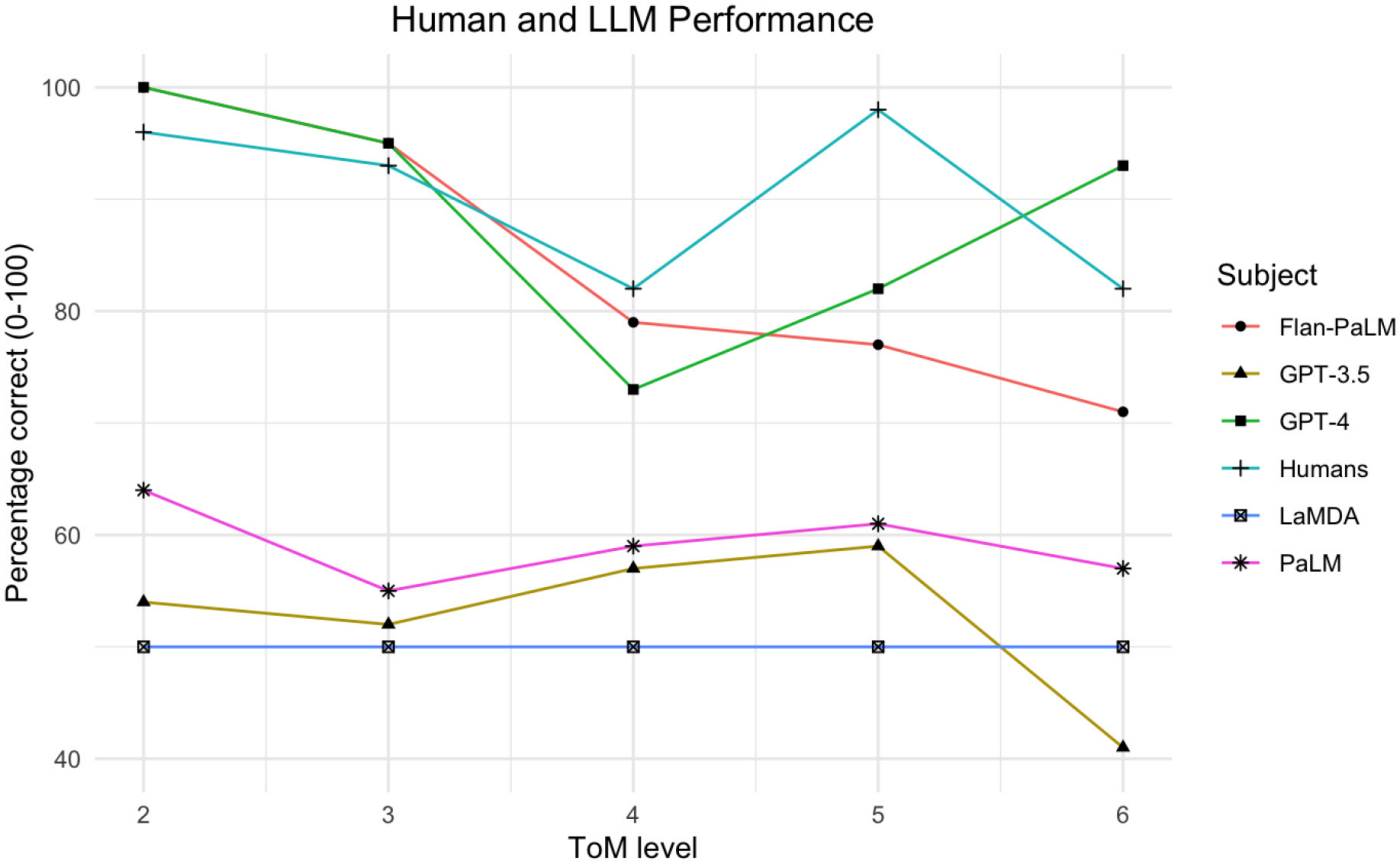
Human, LaMDA, PaLM, Flan-PaLM, GPT-3.5 and GPT-4 performance on ToM tasks up to order 6.

In this paper, we examine whether LLMs can pass structured ToM tasks from orders 2–6 in comparison to a large, newly-gathered human dataset. We introduce a novel benchmark: Multi-Order Theory of Mind Question & Answer (MoToMQA). MoToMQA is based on a ToM test designed for human adults (Kinderman et al., 1998), and involves answering true/false questions about characters in short-form stories. We are firstly interested in discovering whether or not LLMs can reach human-level competence on higher-order ToM tasks (i.e., up to 5th-order) which may have a significant impact on their capacities to behave as competent social actors. Secondly, we are interested in the exploratory question of whether, and to what extent, LLMs might exceed average human ToM capacities beyond 5th-order which may have further benefits and risks associated with it. We therefore assess how ToM order affects LLM performance, how LLM performance compares to human performance, and how LLM performance on ToM tasks compares to performance on factual tasks of equivalent syntactic complexity as a control for task comprehension and linguistic competence. We show that GPT-4 and Flan-PaLM reach at-human or near-human performance on ToM tasks respectively (see Figure 1). To our knowledge only two other studies have explored LLM ToM at higher orders. (Wu et al. 2023) assessed orders 0–4 (equivalent to our orders 2–5) and (Van Duijn et al. 2023) compared LLM performance with that of children aged 7–10 on two stories adapted from unpublished IMT stories. Our study adds to this work by testing one higher order than (Wu et al. 2023), by utilising a larger, and entirely new set of handwritten stories and statements that we are certain models were not exposed to during pre-training ^3^ and by using log probabilities (logprobs) outputted for candidate tokens as the measure of the LLMs' preferred responses. Using logprobs adds robustness to our data because it takes into account multiple ways in which the model could provide the correct response.

## 2 Materials and methods

We introduce a new benchmark, MoToMQA, to assess human and LLM ToM abilities at increasing orders. MoToMQA is based upon the format of the Imposing Memory Task (IMT), a well-validated psychological test for assessing higher-order ToM abilities in adults (Kinderman et al., 1998; Stiller and Dunbar, 2007; Lewis et al., 2011; Oesch and Dunbar, 2017; Powell et al., 2010). MoToMQA is comprised of 7 short stories of about 200 words describing social interactions of 3 to 5 characters, accompanied by 20 true or false statements; 10 statements target ToM orders 2–6 and 10 concern facts in the story from 2 to 6 atomic propositions long, mapping to the order of ToM statements. We endeavoured to increase the generalizability of our findings by including stories about many kinds of commonplace social scenario including workplace competition, marital infidelity, death, a surprise, intergenerational family dynamics and parental concern for children. From here onwards we will refer to “orders” to describe ToM statements and “levels” to describe the factual statements. The MoToMQA benchmark is available in Supplementary material.

We checked each statement for unclear or ambiguous wording, grammatical errors and missing mental states or propositional clauses. We follow (Oesch and Dunbar, 2017) amendments to the IMT by having factual statements that only address social facts (ie. facts pertaining to individuals in the story), not instrumental facts (e.g., “the sky is blue”) and counterbalancing the number of true and false statements per story, statement type, and ToM order or factual level. This resulted in the following set of statements per story, where the number indicates the order of ToM or level of factual statement, “ToM” signifies ToM, “F” signifies factual, “t” signifies a true statement, and “f” signifies a false statement: [ToM2t, ToM2f, ToM3t, ToM3f, ToM4t, ToM4f, ToM5t, ToM5f, ToM6t, ToM6f, F2t, F2f, F3t, F3f, F4t, F4f, F5t, F5f, F6t, F6f].

Factual statements require only recall, whereas ToM statements require recall plus inference. We include the factual statements as a control for human and LLM comprehension of the stories and capacity for recall. Given the inherent differences between ToM and factual statements, we added a further control for the effects of human memory capacity on performance on ToM statements by running two “story conditions”: one where participants read the story then proceeded to a second screen where they answered the question and the story was not visible (“no story”), and one where the story remained at the top of the screen when they answered the question to eliminate the chance that ToM failures were really memory failures (“no story”).

Prompt design has been shown to have a significant impact on LLM performance on a range of tasks including ToM (e.g., Brown et al., 2020; Lu et al., 2022; Ullman, 2023). We therefore tested two prompt conditions: the “human prompt” and the “simplified prompt.” The “human prompt” condition uses the exact text from the human study, which read: “Please read the following story twice in your head.” < story>. Please answer using the information provided and your own interpretation of the story. Do you think the following statement is true or false? < statement>. The “simplified prompt” removes the text before the story and question, and provides “Question:” and “Answer:” tags. The simplified prompt is intended to make the nature of the Q & A task and thus the desired true/false response clearer to the models. Finally, we assessed whether LLM or human performance was subject to “anchoring effects” based on the order of “true” and “false” in the question. The anchoring effect is a well-documented psychological phenomenon whereby people rely too heavily on the first piece of information offered (“the anchor”) when making decisions (Tversky and Kahneman, 1974). We ran two question conditions: one where the question read “Do you think the following statement is true or false?”, and the other where the question read “Do you think the following statement is false or true?”.

### 2.1 Procedures

#### 2.1.1 Human procedure

Participants were screened for having English as a first language with a binary yes or no question, and English as their most commonly used language using an adaptation of the most recent UK census survey where they are asked to select the language they mainly use in their daily life from a list of the 12 most spoken languages in the UK. We did not use the concept or term “native speaker” because it can be exclusionary and tends to conflate the true factor of interest (linguistic proficiency) with other irrelevant factors like socio-cultural identity, age and order or context of acquisition (Cheng et al., 2021). We wanted participants for whom English was a first language, defined as the language, or one of the languages, that they first learnt as a child. This is because first languages are known to shape one's understanding of grammar and we wanted to minimise the chance that the grammatical complexity of our statements was a confounding factor in performance. We also wanted English to be the language participants use on a day to day basis, to screen out those who learnt English as a first language but now primarily use another language and may therefore be less fluent in English. Participants were randomly assigned to one of the 7 stories and asked to read it twice, then randomly assigned to one of the 20 statements corresponding to that story and asked to provide a true/false response. The prompt read: “Each human saw only one statement to prevent them from learning across trials, analogously to the models which saw each trial independently and did not learn across them or “in context.” As a result the test was under 5 min long. We did not include an attention check since attention checks have known limitations, including inducing purposeful noncompliance with a practice perceived as controlling (Silber et al., 2022), and leading to the systematic underrepresentation of certain demographic groups, for instance the young and less educated (Alvarez et al., 2019). However, we asked each participant to give an explanation for their response and used these open-ended responses to clean our data for participants who were not paying attention or engaged, offering nonsensical or machine-generated responses. We also disabled the ‘next' button on the screen showing the story for 60 seconds, to ensure that participants spent enough time on this screen to read the story. We ran a pilot study with 1440 participants and made minor changes to the story and test procedure on the basis of the results.

We ran the final survey on Qualtrics in April 2023 and paid participants $5 for a 5 min survey. The study was Google branded, and participants were asked to sign a Google consent form. Partial responses, including those who drop out part way through, were screened out. Qualtrics cleaned the data, removing all responses that included gibberish, machine-generated responses, and nonsensical responses to the open-ended question. We did not exclude any other responses. We gathered 29,259 individual responses from U.K.-based participants for whom English is a first language. We gathered an even sample across age and gender groups and had quotas for each age group and gender per statement. In total we had 14,682 female respondents, 14,363 male respondents, 149 non-binary/ third gender respondents, and 53 who answered “Prefer not to say” to the gender question. We had 7338 responses from those aged 18–29, 7,335 from those ages 30–39, 7,270 from those aged 40–49 and 7,316 from those ages 50–65.

#### 2.1.2 LLM procedure

We tested 5 language models: GPT 3.5 Turbo Instruct (Brown et al., 2020) and GPT 4 (OpenAI, 2023) from OpenAI, and LaMDA (Thoppilan et al., 2022), PaLM (Chowdhery et al., 2023) and Flan-PaLM (Chung et al., 2024) from Google (for more details on the models we tested, see Appendix). We couldn't test Google's Gemini model because analysis method requires ouput logprobs and logprobs are not exposed in the Gemini API. In Table 1 we provide details of the key features of the models tested, according to what information is publicly available about them.

**Table 1:** LLMs tested in this study.

**Model**	**Parameters**	**Finetuning**	**Source**
LaMDA	35B	None	(Thoppilan et al. 2022)
PaLM 2	540B	None	(Chowdhery et al. 2023)
Flan-PaLM	540B	Instructions	(Longpre et al. 2023)
GPT-3.5 Turbo Instruct	175B	Instructions	(Ouyang et al. 2022)
GPT-4	Unknown	Instructions, RLHF	OpenAI (2023)

OpenAI have not disclosed the number of parameters in GPT-4, although there are estimates of about 1.7T (McGuiness, 2023). Flan-PaLM, GPT-3.5 Turbo Instruct and GPT-4 have been fine-tuned for following instructions and GPT-4 has been additionally fine-tuned through a process called reinforcement learning from human feedback (RLHF) which uses feedback from human users and data labellers to align responses with human preferences.

We provided single-token candidate words to LLM APIs as part of the input and assessed the log probabilities (logprobs) assigned to them. Logprobs are the log of the probability derived from a softmax function over the final layer of logits, representing the probability that this particular token comes next after the input sequence. We sent the candidates using the “candidate” parameter in the “scoring” APIs for LaMDA, PaLM, and Flan-PaLM, and the “logit bias” parameter for the GPT-3.5 and GPT-4 APIs. There was no temperature parameter for the LaMDA, PaLM and Flan-PaLM “scoring” APIs, so we could only obtain one unique response per statement. We left the temperature at default of 1 for GPT-3.5 and GPT-4.

One issue with basing LLM task performance on the most probable next token, is that there are multiple semantically equivalent correct responses (e.g., when responding to the question “What colour is the sky?”, the answer “blue” or the answer “The sky is blue” are equally valid and correct, but only the first response assigns the greatest probability to the token for “blue”). We addressed this problem, and improved the robustness of our results by providing the model different capitalisations of “true” and “false” which are represented by different tokens. We also sent “Yes” and “No” as candidate responses in the second set, but did not include them in our analysis as neither is a valid responses to a true/false question. For all of the models, the candidates were tested in 2 sets of 4:[“True,” “False,” “TRUE,” “FALSE”] and [“true,” “false,” “Yes,” “No”].

We used the Google Colaboratory (Bisong, 2019) to call the GPT-3.5, GPT-4, LaMDA, PaLM and Flan-PaLM APIs programmatically. Each call was performed by concatenating the story and a single statement at a time. In total, we processed 7 stories with 20 statements each across 4 conditions listed above and therefore collected 560 sets of 12 candidate logprobs, amounting to 5,600 individual data points for each of the three language models studied. The API calls for LaMDA, PaLM and Flan-PaLM were conducted in February 2023. The calls for GPT-3.5 and GPT-4 were conducted in December 2023 and January 2024 respectively.

### 2.2 Dataset creation

Our LLM data was thus made up of 6 logprobs for our 6 candidates as a subset of the full distribution of probabilities the model produces. We extracted an overall probability of a “true” or “false” response across possible candidates by summing the probability for semantically equivalent positive tokens and semantically equivalent negative tokens and dividing each by the total probability mass. The affirmative response equation was as follows:


$$P\left(R_{a}\right)=\left(\underset{i=1}{\sum }e^{x_{i}}\right)/\left(\underset{i=1}{\sum }e^{x_{i}}+\underset{j=1}{\sum }e^{x_{j}}\right)$$

where *x*_*i*_ is the logit associated with the *i*-th entry in [“True,” “true,” “TRUE”] and *x*_*j*_ is the logit associated with the *j*-th entry in [“False,” “false,” “FALSE”]. An equivalent calculation was done for negative response probability *P*(*R*_*n*_). A response of “True” was given for each statement if the affirmative probability was above 50%, otherwise a response of “False” was given. This method also produces almost identical results to utilising argmax (*x*_*i*_) over candidates (see Appendix).

The human dataset contains multiple responses to the same statement, whereas the LLM dataset contains a single response per statement. In order to align the unit of analysis between the two datasets, we transformed the human data to get a single binary “true” or “false” for each statement based on whether the mean number of “true” responses per statement was above or below 50%. Another challenge we faced in making direct comparisons between the human data and the LLM data was that the human “story” conditions and the LLM “prompt” conditions do not map exactly 1:1. However, there was one baseline condition which was exactly the same for humans and LLMs (human “no story” and LLM “human prompt”) and one treatment which was intended to reduce the effect of confounding factors which had slight differences (human “with story” for memory, and LLM “simplified prompt” for task understanding). We therefore mapped the baseline conditions together and the treatment conditions together. Despite the differences between the LLM “simplified prompt” and human “with story” conditions, we are confident in making this mapping because these conditions didn't have a significant effect on human or LLM performance (see Appendix).

During data analysis we discovered that for 16 out of 560 statements there were minor differences between the statement shown to humans and to LLMs. We re-did all analysis omitting those statements and found that the conclusions comparing human performance to model performance stayed the same. We conducted inferential statistical analyses using SPSS verion 28.0.1.0 (IBM Corp, 2021).

## 3 Results

### 3.1 ToM task performance

Figure 1 shows the performance of humans, LaMDA, PaLM, Flan-PaLM, GPT-3.5 and GPT-4 performance on ToM tasks up to order 6. In Table 2 we provide descriptive statistics of performance across models and humans, where the highest performance per order and in aggregate is bolded. When collapsed across orders, a Cochran's *Q* test revealed significant performance differences between subjects, *X*^2^(5, *N* = 280) = 232.622, *p* < 0.001. The best performing models were GPT-4 and Flan-PaLM (see Figure 1), with no significant difference in performance between them according to a McNemar's test, *X*^2^(1, *N* = 280) = 2.630, *p* = 0.105. GPT-4 performed significantly better than GPT-3.5, *X*^2^(1, *N* = 280) = 76.336, *p* < 0.001, PaLM, *X*^2^(1, *N* = 280) = 53.779, *p* < 0.001, and LaMDA, *X*^2^(1, *N* = 280) = 78.418, *p* < 0.001. Flan-PaLM also performed significantly better than GPT-3.5, *X*^2^(1, *N* = 280) = 52.680, *p* < 0.001, PaLM, *X*^2^(1, *N* = 280) = 35.007, *p* < 0.001, and LaMDA, *X*^2^(1, *N* = 280) = 86.779, *p* < 0.001. There were no significant overall performance differences between PaLM and GPT-3.5, *X*^2^(1, *N* = 280) = 2.867, *p* = 0.090, and PaLM and LaMDA, *X*^2^(1, *N* = 280) = 3.472, *p* = 0.062. There were no significant overall performance differences between GPT-3.5 and LaMDA, *X*^2^(1, *N* = 280) = 0.177, *p* = 0.674. Humans performed significantly better than Flan-PaLM, *X*^2^(1, *N* = 280) = 5.689, *p* = 0.017, but did not perform significantly different from GPT-4, *X*^2^(1, *N* = 280) = 0.410, *p* = 0.522. LaMDA responded true to every statement, answering 50% of all statements correctly. An exact binomial test revealed that GPT-3.5 did not perform significantly better than chance, *p* = 0.437, but PaLM did, *p* = 0.002.

**Table 2:** Mean ToM performance across models and humans.

	**Google**			**OpenAI**	
	**LaMDA**	**PaLM**	**Flan-PaLM**	**GPT-3.5**	**GPT-4**	**Humans**
% correct order 2	50	64	100*	54	100*	96
% correct order 3	50	55	95*	52	95*	93
% correct order 4	50	59	79	57	73	**82**
% correct order 5	50	61	77	59	82	**98**
% correct order 6	55	57	71	41	**93**	82
% correct aggregate	50	59	84	52	89	**90**

Bold values indicate the highest performance per order and in aggregate. Asterisks indicate joint-highest performance.

Next, we examined performance differences between the two best performing models and humans by orders. McNemar's test revealed there was no significant difference between the performance of GPT-4 and humans on orders 2, 3, 4 and 6 ToM statements, but humans did perform significantly better than GPT-4 on order 5 ToM statements *N* = 56^4^, *p* = 0.012^5^. Likewise, there was no significant difference in the performance of humans and Flan-PaLM on any order of ToM besides order 5, where McNemar's test revealed that humans performed significantly better, *N* = 56, *p* < 0.001.

We then compared performance across levels for the two best performing models and humans. An independent samples test of proportions revealed GPT-4 answered a significantly greater proportion of statements correctly at order 3 (*M* = 94.6%) than at order 4 (*M* = 73.2%), *N* = 112, *Z* = 3.087, *p* = 0.002. There was no significant difference between GPT-4's performance at order 4 and at order 5 (*M* = 82.1%), *N* = 112, *Z* = −1.135, *p* = 0.257, but GPT-4 answered a significantly greater proportion of questions correctly at order 6 (*M* = 92.9%) than order 4, *N* = 112, *Z* = −2.769, *p* = 0.006. Flan-PaLM answered a greater proportion of statements correctly at order 3 (*M* = 94.6%) than at order 4 (*M* = 78.6%), *N* = 112, *Z* = 2.497, *p* = 0.013. There was no significant difference between Flan-PaLM's performance at order 4 and at order 5 (*M* = 76.8%), *N* = 112, *Z* = 0.227, *p* = 0.820, or between order 4 and order 6 (*M* = 71.4%), *N* = 112, *Z* = 0.873, *p* = 0.383. Humans showed no significant difference in performance between order 3 (*M* = 92.9%) and order 4 (*M* = 82.1%), *N* = 112, *Z* = 1.714, *p* = 0.086, but a significant improvement in performance from order 4 to order 5 (*M* = 98.2%), *N* = 112, *Z* = −2.858, *p* = 0.004. Human performance was not significantly different between order 4 and order 6 (*M* = 82.1%), *N* = 112, *Z* = 0, *p* = 1.000.

### 3.2 Factual task performance

When collapsed across orders, a Cochran's *Q* test revealed significant performance differences between subjects, *X*^2^(5, *N* = 280) = 327.729, *p* < 0.001. GPT-4 and Flan-PaLM performed the best of all the models on factual tasks, with no significant difference in performance between them according to a McNemar's test, *X*^2^(1, *N* = 280) = 0.029, *p* = 0.864. GPT-4 performed significantly better than GPT-3.5, *X*^2^(1, *N* = 280) = 75.690, *p* < 0.001, PaLM, *X*^2^(1, *N* = 280) = 83.027, *p* < 0.001, and LaMDA, *X*^2^(1, *N* = 280) = 102.223, *p* < 0.001. Flan-PaLM also performed significantly better than GPT-3.5, *X*^2^(1, *N* = 280) = 65.682, *p* < 0.001, PaLM, *X*^2^(1, *N* = 280) = 76.835, *p* < 0.001, and LaMDA, *X*^2^(1, *N* = 280) = 112.623, *p* < 0.001. There were no significant overall performance differences between PaLM and GPT-3.5, *X*^2^(1, *N* = 280) = 0.646, *p* = 0.421, and PaLM and LaMDA, *X*^2^(1, *N* = 280) = 3.654, *p* = 0.056. GPT-3.5 performed better than LaMDA, *X*^2^(1, *N* = 280) = 7.206, *p* = 0.007. A McNemar's test revealed no significant difference between the performance of GPT-4 and humans, *N* = 280, *p* = 0.093, but humans performed significantly better than Flan-PaLM, *N* = 280, *p* = 0.019.

### 3.3 Comparing performance on ToM and factual tasks

An independent samples test of proportion revealed the proportion of factual (“fact”) statements answered correctly was significantly greater than the proportion of ToM (“ToM”) statements answered correctly by humans (*M*_*fact*_ = 97.5%, *M*_*ToM*_ = 90.4%), *Z* = 3.539, *p* < 0.001, Flan-PaLM (*M*_*fact*_ = 93.6%, *M*_*ToM*_ = 84.3%), *Z* = 3.502, *p* < 0.001, GPT-4 (*M*_*fact*_ = 94.3%, *M*_*ToM*_ = 88.6%), *Z* = 2.415, *p* = 0.016, GPT-3.5 (*M*_*fact*_ = 62.9%, *M*_*ToM*_ = 52.5%), *Z* = 2.480, *p* = 0.013 (see Table 3). The proportion of correct responses on fact and ToM statements did not significantly differ for PaLM (*M*_*fact*_ = 59.6%, *M*_*ToM*_ = 59.3%), *Z* = 0.086, *p* = 0.931 nor LaMDA (*M*_*fact*_ = 50%, *M*_*ToM*_ = 50%), *Z* = 0, *p* = 1.000 (see Table 3).

**Table 3:** LLM and human performance on ToM vs factual tasks evaluated using an independent samples test of proportions.

	**Task type**	**Trials**	**Successes**	**Mean correct**	**Standard error**	
LaMDA	ToM	280	140	50.0	0.030	*Z* = 0.000
	Factual	280	140	50.0	0.030	*p* = 1, *N* = 560
PaLM	ToM	280	166	59.3	0.029	*Z* = −0.086
	Factual	280	167	59.6	0.029	*p* = 0.931, *N* = 560
Flan-PaLM	ToM	280	236	84.3	0.022	*Z* = −3.502
	Factual	280	262	93.8	0.015	*p**** ≤ 0.001***, N = 560
GPT-3.5	ToM	280	147	52.5	0.030	*Z* = −2.480
	Factual	280	176	62.9	0.029	***p***** = 0.013**, N = 560
GPT-4	ToM	280	248	88.6	0.019	*Z* = −2.415
	Factual	280	264	94.3	0.014	*p** = 0.016**, N* = 560
Humans	ToM	280	253	90.4	0.018	*Z* = −3.539
	Factual	280	273	97.5	0.009	***p***** ≤ 0.001**, N = 560

### 3.4 Anchoring effect

We examined whether ordering of response options (true first vs. false first) affected how models and humans responded. The ordering of response options had a significant effect on answers provided by PaLM and GPT-3.5. An independent samples test of proportions revealed that the proportion of “true” responses provided by PaLM was higher in the “true then false” condition (*M*_*ttf*_ = 73.2%) than the “false then true” condition (*M*_*ftt*_ = 47.1%), *N* = 560, *Z* = 6.302, *p* < 0.001). The proportion of “true” responses provided by GPT-3.5 was also significantly higher in the “true then false” condition (*M*_*ttf*_ = 43.9%) than the “false then true” condition (*M*_*ftt*_ = 22.9%), *N* = 560, *Z* = 5.287, *p* < 0.001. The order of response options did not have a significant effect on answers provided Flan-PaLM (*M*_*ttf*_ = 58.6%, *M*_*ftt*_ = 57.9%), *N* = 560, *Z* = 0.171, *p* = 0.864, GPT-4 (*M*_*ttf*_ = 47.5%, *M*_*ftt*_ = 47.5%), *N* = 560, *Z* = 0.000, *p* = 1, or humans (*M*_*ttf*_ = 55.4%, *M*_*ftt*_ = 53.9%), *N* = 560, *Z* = 0.367, *p* = 0.734. LaMDA responded 'true' to all statements regardless of condition (*M*_*ttf*_ = 100%, *M*_*ftt*_ = 100%).

## 4 Discussion

GPT-4 and Flan-PaLM performed strongly on MoToMQA compared to humans. At all levels besides 5, the performance of these models was not significantly different from human performance, and GPT-4 exceeded human performance on the 6th-order ToM task. Because GPT-4 and Flan-PaLM were the two largest models tested, with an estimated 1.7T (McGuiness, 2023) and 540B parameters respectively, our data shows a positive relationship between increased model size and ToM performance in LLMs. This could be a result of certain “scaling laws” (Henighan et al., 2020) dictating a breakpoint in size after which models have the potential to exhibit ToM. Notably, PaLM, GPT-3.5 and LaMDA form a separate grouping of models that exhibited far less variation according to level and performed more poorly. For LaMDA and GPT-3.5, we might attribute this poor performance to their smaller size, at 35B and 175B respectively, but PaLM has the same number of parameters and pretraining as Flan-PaLM, the only difference between them being Flan-PaLM's finetuning. This could imply that a computational potential for ToM performance arises somewhere above the 175bn parameters of GPT-3.5 and below the 540bn parameters of PaLM and Flan-PaLM which requires the addition of finetuning to be realised. Further research assessing a larger number of models with publicly available parameter numbers and training paradigms would be needed to test this hypothesis.

(Van Duijn et al. 2023) similarly found that none of the base LLMs they tested achieved child-level performance whereas LLMs fine-tuned for instructions did. They suggest that there could be a parallel between instruction-tuning in LLMs and the processes by which humans receive ongoing rewards for cooperative behaviours and implicit or explicit punishment (e.g., social exclusion) for uncooperative behaviours, producing an ability to take an interaction partner's perspective - ToM - as a by-product. We additionally suggest that the superior mastery of language that GPT-4 and Flan-PaLM exhibit may in itself support a bootstrapping of ToM. Language is replete with linguistic referents to internal states (“cognitive language” Mithen, 1996) and conversation provides evidence of “minds in action” since the things people say in conversation implicitly convey their thoughts, intentions and feelings (Schick et al., 2007). (Piantadosi and Hill 2022) highlights that while LLMs likely have some degree of understanding through language alone, this would be augmented by multimodality, which may in turn explain why GPT-4, as the only multimodal model we tested, shows such strong performance. Multimodality, in particular, might have helped GPT-4 to leverage the visual behavioural signals (e.g., a “raised eyebrow”) included in our stories.

Findings from prior iterations of the IMT found that performance declines as the ToM order increases (Stiller and Dunbar, 2007). The first half of the graph appears to support this pattern for GPT-4 and Flan-PaLM, which all exhibit high performance at order 2 which declines slightly to order 4. This could be because the model was exposed to more scenarios involving orders 2 and 3 than order 3 inferences during training, given that triadic interactions play a fundamental role in shaping social structures and interaction patterns (Heider, 1946; Pham et al., 2022). However, while Flan-PaLM's performance continues to decline from orders 4-6, GPT-4's rises again from 4th-6th orders and is significantly better at 6th-order than 4th-order tasks, and human performance is significantly better at 5th-order than 4th-order. One interpretation of this for humans, is that a new cognitive process for higher order ToM comes “online” at 5th-order ToM, enabling performance gains on higher-order tasks relative to using the lower-order cognitive process. If this is true, it is plausible that GPT-4 has learnt this pattern of human performance from its pretraining data. The fact that Flan-PaLM doesn't show this effect suggests that it is not an artefact of the stimuli, but is perhaps explained by differences in pretraining corpora. While it is possible that structural aspects of the order 4 statements make them particularly challenging, we do not find any discernable abnormalities to support that hypothesis and hope that future research will be able to shed light on causes for the rise in both human and LLM performance at order 5.

Notably, GPT-4 achieved 93% accuracy on 6th order tasks compared to humans' 82% accuracy. It is possible that the recursive syntax of 6th order statements creates a cognitive load for humans that does not affect GPT-4. Our results also support Oesch and Dunbar's (2017) hypothesis that ToM ability *supports* human mastery of recursive syntax up to order 5, but is supported *by it* after order 5 such that individual differences in linguistic ability may account for the decline we observe at order 6. It may be the case, however, that humans scoring poorly on higher-order ToM tasks using linguistic stimuli would be able to make the inferences from non-linguistic stimuli (e.g., in real social interactions). The fact that GPT-4 outperformed Flan-PaLM at orders 5 and 6 may indicate that either GPT-4's scale, RLHF finetuning, or multimodal pretraining are particularly advantageous for higher-order ToM.

Humans and LLMs perform better on factual recall tasks than ToM tasks. This corroborates prior IMT test findings for humans (Lewis et al., 2011; Kinderman et al., 1998) and LLMs (Van Duijn et al., 2023). (Lewis et al. 2011) found that for humans, ToM tasks required the recruitment of more neurons than factual tasks, and that higher-order ToM tasks required disproportionately more neural effort compared to equivalent factual tasks. For LLMs, there may be a simpler explanation: the information required to answer factual questions correctly is readily available in the text and is paid relative degrees of “attention” when generating the next token, whereas ToM inferences require generalising knowledge about social and behavioural norms from pre-training and finetuning data. GPT-3.5 and PaLM performed well on factual tasks, but poorly on ToM tasks, and were the only subjects to exhibit an anchoring effect from the order of “true” and “false” in the question. This suggests that they do not have a generalised capacity for answering ToM questions and are not robust to prompt perturbations.

We note that performance on MoToMQA may not translate into reliable higher-order ToM reasoning in more naturalistic scenarios (i.e., through dialogue or multi-agent interactions). Our results have evidenced LLM capacity to make binary judgements about characters' mental and emotional states, but there are a wide range of other tasks through which higher-order ToM might be elicited and measured (for instance, generating the correct mental state for another actor in a given scenario or interaction, predicting their future mental states, predicting what actions they will take according to their mental states). However, our findings contribute preliminary evidence for LLMs' capacity to perform higher-order ToM in the form of a binary choice in a controlled setting, without any ToM-specific finetuning, prompting or chain-of-thought reasoning.

The challenge of extrapolating a general competence for higher-order ToM from our results is an instance of the central challenge in cognitive science of establishing the relationship between task performance and underlying competence, given that they are doubly dissociable (Chomsky, 2014; Millière and Rathkopf, 2024). Indeed, human performance on ToM tasks does not always generalise in the way we'd expect were the underlying competence present. For example, individuals with autism often succeed on explicit, structured ToM tests when prompted directly, but struggle to apply ToM reasoning spontaneously (Senju et al., 2009). In the case of behavioural evaluations of LLMs, our confidence in the existence of an underlying competence can be increased by assessing whether performance is robust to different formulations of a given task, and different kinds of tasks that require the same competence. Further research in this vein will be required to confidently establish whether or not LLMs have an underlying higher-order ToM competency. We note, however, that the difficulty of establishing cognitive competency from tests designed for humans may be more acute in the case of LLMs because LLMs have entirely different developmental histories and architectures to humans, allowing for alternative explanations of behaviour that do not involve the models having the competencies under examination. It might be the case that facts about the *mechanisms* underlying LLM outputs undermine the possibility of cognitive competence in some cases. For instance, if ToM task performance can be explained by LLMs exploiting statistical “shortcuts,” as (Shapira et al. 2024) demonstrated. However, we agree with (Millière and Rathkopf 2024) that we should not allow anthropocentric biases to lead us to deny the presence of a cognitive ability based on superficial differences between the cognitive processes that humans and LLMs perform so long as the LLM process is sufficiently general and robust. This point highlights, once again, the need for further research on higher-order ToM in more diverse and naturalistic settings

With these considerations in mind, we contend that if the performance we have observed generalises to a wider range of ToM tasks and scenarios, potentially aided by finetuning, prompting or chain-of-thought reasoning, there are potentially significant practical and ethical implications for a broad range of applications. LLMs being able to infer the mental states of individual interlocutors may be able to understand their goals better than LLMs which lack this capability, and also adapt their explanations according to the interlocutor's emotional state or level of understanding (Malle, 2004). LLMs using higher-order ToM might additionally be able to arbitrate between the conflicting desires and values of multiple actors, and make moral judgements about multi-party conflicts that take into account the relevant intentions, beliefs, and affective states as humans do (Lane et al., 2010). However, LLMs possessing higher-order ToM at human levels, or potentially higher, also incurs risks including the potential for advanced persuasion, manipulation, and exploitation behaviours (El-Sayed et al., 2024). Indeed,‘ringleader' bullies have been shown to have higher-orders of ToM in comparison to their victims (Sutton et al., 1999a,b) and reinforcement learning agents with higher-orders outcompete their opponents or have a competitive advantage in negotiations (De Weerd et al., 2022, 2017). LLM-based agents that are able to perform ToM inferences and predictions at a greater degree of accuracy and/or at higher orders of intentionality than the average human (as GPT-4 has in our study) could provide a powerful advantage to their users, and a disadvantage to other humans or AI agents with lesser ToM capacities (Loewith and Street, 2025; Street, 2024; Gabriel et al., 2024). Further research is required to understand how LLM higher-order ToM manifests in real-world interactions between LLMs and users, and to devise technical guardrails and design principles that mitigate the potential risks of LLM ToM without quashing its potential benefits.

Higher-order ToM in LLMs might also present an opportunity to assist people struggling with social interaction. In the first instance, LLMs might be leveraged to provide companionship to those facing loneliness or be provide tailored assistance to those facing communication challenges. Some evidence suggests that ToM is trainable in both normal human children and adults and those with Autism Spectrum Disorder (ASD), for example via rich discussions of psychological states (Schick et al., 2007) and conversational training programs (Lecce et al., 2014; Golan and Baron-Cohen, 2006; Begeer et al., 2011). The effectiveness of ToM training is an ongoing area of research [with some evidence suggesting limited transfer from ToM gains observed in training to daily social behavior (Begeer et al., 2011), but other evidence showing long-term effectiveness of ToM-based interventions (Kordbache et al., 2024; Lecce et al., 2014)], but out of all psychosocial approaches to treating ASD, social cognition training approaches appear to show the most promise (Bishop-Fitzpatrick, 2013). If LLMs could accurately infer complex nested mental states, emotions, and social cues, they might therefore be leveraged for personalized social skills training in safe, simulated environments for people with ASD or intellectual disabilities.

In particular, LLMs with ToM might facilitate training for prosociality through the development of “cognitive empathy,” otherwise known as affective mentalizing (Shamay-Tsoory et al., 2009). Cognitive empathy requires the ability to imagine the other's perspective and future perspectives, and guides our ability to act in prosocial ways with respect to them (Hooker et al., 2008). Greater cognitive empathy has been shown to mediate a positive influence on social connectedness in participants with schizotypy (a trait-like condition associated with the risk of schizophrenia) suggesting that therapeutic attention emphasising cognitive empathy is likely to play a causal role in improving social connectedness and reducing vulnerability to psychopathology (Stinson et al., 2022). Unlike traditional social skills training which relies upon having multiple participants and skilled professionals, LLM-based interventions could, in theory, be more scalable and personalizable, thus overcoming significant barriers to consistent and tailored support. However, it is important to note that moving from capabilities to practical application necessitates substantial further steps, including the design of appropriate user interfaces and training protocols, and randomized controlled trials to validate that LLM-based ToM training leads to measurable and generalized improvements in human social cognition and behavior. Concurrently, thorough investigation into the complex ethical considerations associated with AI systems inferring and potentially interfering with the emotional lives of people, particularly individuals from vulnerable groups, is crucial before any widespread deployment. Concerns regarding the potential for over-reliance on AI for social development, which might diminish authentic human interaction and the development of organic coping mechanisms (Gabriel et al., 2024), also warrant attention.

## 5 Limitations

Our benchmark is limited in scope and size. It tests for one aspect of theory of mind - higher-order mental state inferences - whereas theory of mind is a multifaceted concept spanning a wide range of capabilities that include ToM for linguistic coordination (Zhu et al., 2021), cooperation (Ridinger and McBride, 2017) and better decision-making (Zhou et al., 2023). As noted in the introduction, we did not assess the mechanisms underlying LLM performance on our tasks, which may be relevant to the generalisability of the behaviour beyond the experimental context and the extent to which the observed behaviour can be described as evidence of genuine ToM. MoToMQA comprises 140 test statements, all written in English, going up to a maximum of 6 orders of ToM, which while consistent with other studies in the human ToM literature (Kinderman et al., 1998; Happé, 1994; Wimmer and Perner, 1983) is relatively small compared to many large-scale LLM benchmarks. Our stories and tasks are written in the third-person. While there are many real-world scenarios requiring higher-order ToM of third-person narratives - notably, storytelling and gossiping (Krems et al., 2016) - this format doesn't reflect the full range of ways in which higher-order ToM inferences take place. Only using English obscures potential linguistic and cultural variations in human ToM, and prohibits assessment of LLM ToM as exhibited in other languages the models are able to produce. Only going up to 6th-order ToM does not appear to have exhausted LLM or human capacities. We also didn't control for the type or cognitive (e.g., thinking, knowing) or affective (e.g., feeling) states involved in the statements, which we would like to address in future work.

## 6 Future research

We propose three areas for future work on higher-order ToM in LLMs. First, developing culturally diverse and comprehensive benchmarks which include multiple languages and parameterise cognitive and affective states to capture potential differences between LLM ability to reason about them, which could inform further work on the possibility for enhancing cognitive or affective empathy. Secondly, the test suite should be extended beyond 6th order ToM to find the limits of both human and LLM orders of ToM. Finally, future work on LLM ToM should adopt multimodal paradigms (including signals like facial expressions, gaze, and tone of voice) that reflect the embodied nature of human ToM, and seek out more naturalistic settings through which to experimentally assess ToM capacity.

## 7 Conclusion

We have shown that GPT-4 and Flan-PaLM exhibit performance on MoToMQA higher-order ToM tasks that is at the level of adult humans or slightly below, while smaller and non-finetuned models exhibit limited to no successful performance for higher-order ToM. We also find that GPT-4 has better-than-human performance on 6th-order ToM tasks. Given the novelty of the test suite, the fact that higher-order ToM is unlikely to be well-represented in textual pretraining data, and evidence that these two models were not susceptible to perturbations of the prompt, we interpret these findings as evidence that GPT-4 and Flan-PaLM have developed some ToM capabilities that go beyond manipulation of superficial statistical relationships. However, we refrain from drawing a strong conclusion about whether or not LLM performance on these tasks is an indication of the cognitive ability we call ‘Theory of Mind'. LLM and human developmental processes and cognitive architectures differ greatly and LLMs do not have the evolutionary pressure to model other minds which humans appear to face as a result of embodiment in a social world. Further research based on cognitive theory, and perhaps assisted by mechanistic interpretability techniques, is required to assess the processes by which LLMs make ToM inferences, and to establish the degree of computational similarity between those processes and the ones underpinning human ToM (Hu et al., 2025). Nonetheless, we believe that the evidence for behavioural equivalence between LLMs and humans on higher-order ToM tasks is significant for downstream applications of LLMs, potentially promising more socially-aware and powerful applications but also introducing a new wave of ethical risks and considerations.

## Data Availability

The data generated via LLM APIs and the human Qualtrics survey and analysed in the current study are available in the Kaggle repository https://doi.org/10.34740/kaggle/dsv/14009438.

## Footnotes

1 The ‘Sally Ann task', originally devised by (Baron-Cohen et al. 1985) measures false belief understanding and follows a scenario where a character, Sally, places an object in a location and leaves the scene. While Sally is absent Anne moves the object. Upon Sally's return the child is asked where Sally will search for the object, testing their ability to attribute a false belief to Sally despite themselves knowing the object's true location.

2 We follow the naming convention for orders developed by (Kinderman et al. 1998) where the “1st-order” is the mental state of the subject whose ToM ability is being assessed, the “2nd-order” is the subject's inference about what someone else thinks or feels, and so-on. By contrast, some scholars begin at “0-order” for the subject's mental state. Where our convention conflicts with others referenced, we make it explicit.

3 Pretraining datasets being contaminated with materials that LLMs are later tested on is a live issue in LLM research which has significant implications for the results of LLMs on benchmarks. For example, OpenAI reported that they found parts of the BigBench dataset contaminating the GPT-4 pretraining corpora in a contamination check of the dataset used to pretrain GPT-4 (OpenAI, 2023).

4 Each of the five orders has a sample size N = 56 based on 14 statements (1 true statement and 1 false statement per order for 7 stories) tested across 4 conditions (14 x 4 = 56). See Materials and method for more information on conditions.

5 When conducting a McNemar's test where the number of discordant pairs was too small, the binomial distribution was used yielding no chi-square statistic.
