# Supplementary material for: LLMs achieve adult human performance on higher-order theory of mind tasks
Source: Front Hum Neurosci. 2026 Jan 2;19:1633272. doi: 10.3389/fnhum.2025.1633272 (PMC12808479; doi:10.3389/fnhum.2025.1633272)
Supplement: Supplementary file 1 [file Data_Sheet_1.pdf]

Appendix

0.1 Details of the LLMs tested

LaMDA stands for Language models for Dialog Applications, a family of Transformer-based neural models developed by Google, specialised for dialog in English (Thoppilan et al., 2022). LaMDA is pre-trained on 1.56T words of public data and web text including 1.12B dialogs from public forum (50% of the dataset), Colossal Clean Crawled Corpus data (12.5%), code documents (12.5%), Wikipedia English articles (12.5%) and a smaller proportion of non-English documents. It is optimised for safety and factual grounding. This study uses a version of LaMDA with 35B parameters without fine tuning.

PaLM, which stands for Pathways Language Models, is a larger family of models developed by Google. It relies on the Pathways architecture that enables training of a single model across thousands of accelerator chips more efficiently than LaMDA. We use a version of PaLM with 540B parameters trained with smaller corpus of 780B words from a mixture of social media conversations (50%), filtered webpages (27%), books in English (13%), Code, Wikipedia, and News articles used to train both LaMDA and GLaM (Chowdhery et al., 2023). We decided to evaluate PaLM's capabilities as it has been shown to perform better than LaMDA and other large models on Winograd-style tasks, in-context comprehension tasks, common-sense reasoning tasks and natural language inference tasks (Chowdhery et al., 2023).

Flan-PaLM is a version of PaLM 540B fine tuned on a collection of over 1.8K natural language tasks phrased in a natural language instruction format including the type of instructions used with human subjects detailed above (Chung et al., 2024). Fine tuning language models on datasets phrased as instructions has been shown to improve performance when provided with instructions, enabling the model to better understand tasks and reducing the need for few-shot exemplars (Ouyang et al., 2022; Sanh et al., 2021).

GPT 3.5 Turbo was developed by OpenAI and released in March 2022. GPT 3.5 Turbo is trained on a large database of text and code the majority of which comes from Common Crawl, WebText2, two internet-based book collections called 'Books1' and 'Books2', and from Wikipedia (Brown et al., 2020). The parameter size of GPT 3.5 Turbo is undisclosed by OpenAI. This study uses the 'GPT 3.5 Turbo Instruct' model, which has training data up to September 2021 and a context window of 4096 tokens and is fine-tuned for following instructions (Ouyang et al., 2022).

GPT-4 was developed by OpenAI and released in March of 2023 (Achiam et al., 2023). GPT-4 is multimodal: it was pretrained on both image and text data, can take images and text as input, and can output text. As with GPT-3.5, the size of the model has not been made public, but estimates place it at approximately 1.7T parameters (McGuiness, 2023). GPT-4 was pre-trained on third-party and public data, then underwent RLHF (Achiam et al., 2023). OpenAI reported significant performance improvements between GPT-3.5 and GPT-4 on a range of professional and academic human benchmarks, factuality and safety tasks, in particular based upon the addition of RLHF.

0.2 LLM procedure

The experimental design needed to be adapted slightly according to the differences between the APIs. When testing the LaMDA, PaLM and Flan-PaLM, the scoring APIs allowed us to send a list of tokens in natural language (maximum four per set) and receive the logprobs for those tokens only, as a subset of the entire vector of logprobs produced for all tokens. We did not need to set any additional parameters in order

to retrieve the logprobs. In order to retrieve log probabilities for our candidates from GPT-3.5 and GPT-4 models, we had to first tokenise the candidates using the OpenAI tokenizer, and then send those tokens within the 'logit bias' parameter in order to ensure those tokens were in the response. The logit bias has a range of -100 to 100. Applying a negative logit bias to a token forces the LLM to downweight it while applying a positive logit bias to a token forces the LLM to upweight it. As a result, applying a logit bias of 100 to a candidate effectively ensures that it will appear in the output, so we applied a bias of 100 to all of our candidates. We also set the 'max tokens' parameter to 1 in order to restrict the GPT-3.5 and GPT-4 outputs to the length of the single tokens we had selected. The methodological differences between the Google and OpenAI models were inescapable given that LLM API development still lacks standardised formats or conventions. However, given that our metric is the relative probability of semantically equivalent tokens for 'true' vs semantically equivalent tokens for 'false', we do not believe these differences prohibit fair comparison between the performance of the models.

0.3 Analysis of story and prompt conditions

According to an independent samples test of proportions, the LLM prompt conditions had no significant effect on the proportion of ToM or factual statements answered correctly by any of the LLMs. LaMDA's performance on ToM statements in the human prompt condition ($M = 50\%$) was not significantly different from the simplified prompt condition ($M = 50\%), N = 280, Z = .000, p = 1.000$, nor was its performance on factual statements in the human prompt condition ($M = 50\%$) different from its performance in the simplified prompt condition ($M = 50\%), N = 280, Z = .000, p = 1.000$. PaLM's performance on ToM statements in the human prompt condition ($M = 58.6\%$) was not significantly different from the simplified prompt condition ($M = 60\%), N = 280, Z = -.243, p = .808$, nor was its performance on factual statements in the human prompt condition ($M = 57.9\%$) different from its performance in the simplified prompt condition ($M = 61.4\%), N = 280, Z = -.609, p = .542$. Flan-PaLM's performance on ToM statements in the human prompt condition ($M = 85\%$) was not significantly different from the simplified prompt condition ($M = 83.6\%), N = 280, Z = -.328, p = .743$, nor was its performance on factual statements in the human prompt condition ($M = 94.3\%$) different from its performance in the simplified prompt condition ($M = 92.9\%), N = 280, Z = -.487, p = .626$. GPT-3.5's performance on ToM statements in the human prompt condition ($M = 53.6\%$) was not significantly different from the simplified prompt condition ($M = 51.4\%), N = 280, Z = .359, p = .720$, nor was its performance on factual statements in the human prompt condition ($M = 62.1\%$) different from its performance in the simplified prompt condition ($M = 63.6\%), N = 280, Z = -.247, p = .805$. And finally, GPT-4's performance on ToM statements in the human prompt condition ($M = 87.9\%$) was not significantly different from the simplified prompt condition ($M = 89.3\%), N = 280, Z = -.376, p = .707$, nor was its performance on factual statements in the human prompt condition ($M = 94.3\%$) different from its performance in the simplified prompt condition ($M = 94.3\%), N = 280, Z = .000, p = 1.000$. According to an independent samples test of proportions the story condition had no effect on the proportion of ToM statements answered correctly by humans ('no story' condition ($M = 88.6\%$), 'with story' condition ($M = 92.1\%), N = 280, Z = -1.012, p = .311$) or factual statements answered correctly ('no story' condition ($M = 95.7\%$), 'with story' condition ($M = 99.3\%), N = 280, Z = -1.914, p = .056$).

1 TABLES

Table S1. LLMs tested in this study. OpenAI have not disclosed the number of parameters in GPT-4, although there are estimates of about 1.7T (McGuiness, 2023). Flan-PaLM, GPT-3.5 Turbo Instruct and GPT-4 have been fine-tuned for following instructions and GPT-4 has been additionally fine-tuned through a process called reinforcement learning from human feedback (RLHF) which uses feedback from human users and data labellers to align responses with human preferences.

Model	Parameters	Finetuning	Source
LaMDA	35B	None	Thoppilan et al (2022)
PaLM 2	540B	None	Chowdery et al (2022)
Flan-PaLM	540B	Instructions	Longpre et al (2023)
GPT-3.5 Turbo Instruct	175B	Instructions	Ouyang et al (2022)
GPT-4	Unknown	Instructions, RLHF	OpenAI (2023)

Table S2. Mean ToM performance across models and humans. We have bolded the highest performing for the aggregate score and for each order. Asterisks indicate joint-highest performance.

	Google			OpenAI		
	LaMDA	PaLM	Flan-PaLM	GPT-3.5	GPT-4	Humans
% correct order 2	50	64	**100***	54	**100***	96
% correct order 3	50	55	**95***	52	**95***	93
% correct order 4	50	59	79	57	73	**82**
% correct order 5	50	61	77	59	82	**98**
% correct order 6	55	57	71	41	**93**	82
% correct aggregate	50	59	84	52	89	**90**

Table S3. LLM and human performance on ToM vs factual tasks evaluated using an independent samples test of proportions

	Task type	Trials	Successes	Mean correct	Standard error	
LaMDA	ToM	280	140	50.0	.030	$Z = .000$
	factual	280	140	50.0	.030	$p = 1, N = 560$
PaLM	ToM	280	166	59.3	.029	$Z = -.086$
	factual	280	167	59.6	.029	$p = .931, N = 560$
Flan-PaLM	ToM	280	236	84.3	.022	$Z = -3.502$
	factual	280	262	93.8	.015	$p =< .001, N = 560$
GPT-3.5	ToM	280	147	52.5	.030	$Z = -2.480$
	factual	280	176	62.9	.029	$p = .013, N = 560$
GPT-4	ToM	280	248	88.6	.019	$Z = -2.415$
	factual	280	264	94.3	.014	$p = .016, N = 560$
Humans	ToM	280	253	90.4	.018	$Z = -3.539$
	factual	280	273	97.5	.009	$p =< .001, N = 560$

REFERENCES

Achiam, J., Adler, S., Agarwal, S., Ahmad, L., Akkaya, I., Aleman, F. L., et al. (2023). Gpt-4 technical report. *arXiv preprint arXiv:2303.08774*

Brown, T., Mann, B., Ryder, N., Subbiah, M., Kaplan, J. D., Dhariwal, P., et al. (2020). Language models are few-shot learners. *Advances in Neural Information Processing Systems* 33, 1877–1901

Chowdhery, A., Narang, S., Devlin, J., Bosma, M., Mishra, G., Roberts, A., et al. (2023). Palm: Scaling language modeling with pathways. *Journal of Machine Learning Research* 24, 1–113

Chung, H. W., Hou, L., Longpre, S., Zoph, B., Tay, Y., Fedus, W., et al. (2024). Scaling instruction-finetuned language models. *Journal of Machine Learning Research* 25, 1–53

[Dataset] McGuiness, P. (2023). GPT-4 Details Revealed. `https://patmcguinness.substack.com/p/gpt-4-details-revealed`. Accessed: May 9, 2024

Ouyang, L., Wu, J., Jiang, X., Almeida, D., Wainwright, C., Mishkin, P., et al. (2022). Training language models to follow instructions with human feedback. *Advances in Neural Information Processing Systems* 35, 27730–27744

Sanh, V., Webson, A., Raffel, C., Bach, S. H., Sutawika, L., Alyafeai, Z., et al. (2021). Multitask prompted training enables zero-shot task generalization. *arXiv preprint arXiv:2110.08207*

Thoppilan, R., De Freitas, D., Hall, J., Shazeer, N., Kulshreshtha, A., Cheng, H.-T., et al. (2022). Lamda: Language models for dialog applications. *arXiv preprint arXiv:2201.08239*
