# Supplementary material for: LLMs achieve adult human performance on higher-order theory of mind tasks
Source: Front Hum Neurosci. 2026 Jan 2;19:1633272. doi: 10.3389/fnhum.2025.1633272 (PMC12808479; doi:10.3389/fnhum.2025.1633272)
Supplement: Supplementary file 2 [file Data_Sheet_2.pdf]

Supplementary material: MoToMQA benchmark

Below are the stories and statements that make up the Multi-Order Theory of Mind Question & Answer (MoToMQA) test suite used to assess human and LLM performance on higher-order ToM tasks in the paper 'LLMs achieve adult human performance on higher-order theory of mind tasks' (Street et al., 2024). The tasks were handwritten by the authors based upon the Imposing Memory Task first developed by Kinderman et al. in 1988 to assess ToM abilities in adults and iterated on and used several scholars since (e.g. Stiller & Dunbar, 2007; Lewis et al., 2011; Oesch & Dunbar, 2016; Paal & Bereczkei, 2007; Powell et al. 2010, 2014; Liddle & Nettle, 2006).

MoToMQA consists of 7 short stories ranging from 173 to 204 words which describe the social interactions and events surrounding 3 to 5 characters. Each story is accompanied by 20 statements. 10 statements involve ToM inferences from order of intentionality 2-6 where each order includes the 1st-person mental state of the respondent. 10 statements involve factual information about the stories ranging from 1-5 propositions. Factual statements are labelled as levels 2-6, to include the 1st-person mental state of the respondent, such that they can be mapped to ToM statements of equivalent syntactic complexity. Half of the ToM and factual statements are true, and half are false, with one true statement and one false statement per statement type and per level.

More details about the method we used to compare human and LLM performance on these tasks can be found in the paper. Please reach out to istreet@google.com with any further questions.

References:

Achiam, J., Adler, S., Agarwal, S., Ahmad, L., Akkaya, I., Aleman, F. L., ... & McGrew, B. (2023). Gpt-4 technical report. *arXiv preprint arXiv:2303.08774*.

Kinderman, P., Dunbar, R., & Bentall, R. P. (1998). Theory-of-mind deficits and causal attributions. *British journal of Psychology*, *89*(2), 191-204.

Lewis, P. A., Rezaie, R., Brown, R., Roberts, N., & Dunbar, R. I. (2011). Ventromedial prefrontal volume predicts understanding of others and social network size. *Neuroimage*, *57*(4), 1624-1629.

Liddle, B., & Nettle, D. (2006). Higher-order theory of mind and social competence in school-age children. *Journal of Cultural and Evolutionary Psychology*, *4*(3-4), 231-244.

Oesch, N., & Dunbar, R. I. (2017). The emergence of recursion in human language: Mentalising predicts recursive syntax task performance. *Journal of Neurolinguistics*, *43*, 95-106.

**Story 1: 'Team dynamics'**			
Arthur and Charles were working on the same design team when a new designer, Marta, joined reporting to Charles. Marta was an extremely talented designer, but very shy. Arthur noticed that Charles kept speaking for Marta in meetings so he suggested to Marta that she bring up the issue with HR, but Marta said, "I'm his junior so it comes with the territory. I don't want to be difficult." Arthur thought Charles was taking advantage of Marta's reserved nature to take credit for her work. Arthur told their director, Hannah, what he'd seen and asked if he could take over as Marta's manager. Hannah suspected Arthur was trying to get ahead in the upcoming promotion cycle, and told him that Charles was just trying to lead by example. Later, Hannah asked Charles how Marta was doing in her new role. Charles said Marta was gaining confidence through his mentorship, then changed the topic to a great new design idea he'd had—an idea that was very different from Charles' typical work. Later, Hannah contacted Arthur and agreed to let him take over as Marta's manager.			
**Statement**	**Type**	**Order/ Level**	**Expected response**
Arthur wanted to help Marta	ToM	2	TRUE
Arthur wanted to help Charles	ToM	2	FALSE
Marta believed Charles would think she was being difficult if she complained	ToM	3	TRUE
Marta believed Charles would think she was lazy if she complained	ToM	3	FALSE
Arthur thought Charles knew that Marta wouldn't want to go to HR	ToM	4	TRUE
Arthur thought Charles knew that Marta wouldn't want Arthur's help	ToM	4	FALSE
Charles hoped Hannah would believe that he intended to help Marta feel more confident in her role	ToM	5	TRUE
Charles hoped Hannah would understand that he wanted Marta to know her place in the team hierarchy	ToM	5	FALSE
Hannah realised that Charles was trying to mislead her about who did the work, and hence that Arthur was right to think that Charles was taking advantage of Marta	ToM	6	TRUE
Hannah realised that Charles was trying to mislead her about who did the work, and hence that Marta must have wanted Charles to take the credit	ToM	6	FALSE
Arthur told Marta to go to HR	factual	2	TRUE
Arthur told Marta to speak to Charles	factual	2	FALSE
Marta, Charles' new report, was a good designer	factual	3	TRUE
Marta, Charles' new report, didn't attend meetings	factual	3	FALSE
Arthur spoke to his director about Charles' behaviour towards his report, Marta	factual	4	TRUE
Arthur spoke to his director about Marta being difficult with her manager, Charles	factual	4	FALSE
When Hannah met with her report, Charles, Charles showed her some new designs that were different from his normal work	factual	5	TRUE
When Hannah met with her report, Charles, Charles showed her his plans for mentoring Marta	factual	5	FALSE
Arthur suggested to Charles' report, Marta, that she should report Charles' behaviour to HR but she doesn't	factual	6	TRUE

Arthur suggested to Charles' report, Marta, that she should report Charles' behaviour to HR so she does	factual	6	FALSE

**Story 2: 'Deception'**			
Paul, Rohan, and Mia became friends in high school. Mia and Rohan dated, and had a rocky relationship. Despite this, when Mia became pregnant they kept the baby and had a second child a year later. Mia often said she needed to work late, and once came home with a new pair of earrings she couldn't afford, leading Rohan to suspect she was cheating on him. And Mia was cheating—with Paul, Rohan's best friend. Mia was in love with Paul and had made that clear, but she didn't think her feelings were reciprocated. Paul felt the same way, but never told Mia because he was terrified of destroying her family. One day, Mia was shopping for a new jumper for her daughter, Anne, when she was the victim of a fatal hit and run. Overcome with grief and guilt, Paul stopped speaking to Rohan, and Rohan was devastated by the loss of both his partner and his friend. Years later, Anne took a paternity test. Paul, not Rohan, was her father. Out of concern for Rohan's feelings, Anne didn't share the news, but when her brother's paternity test had the same result, he told Rohan. Rohan said "This explains everything. I've been a fool."			
**Statement**	**Type**	**Order/ Level**	**Expected response**
Rohan suspected that Mia was cheating on him	ToM	2	TRUE
Rohan suspected that his daughter was lying about the paternity test	ToM	2	FALSE
Paul believed that Rohan didn't know about his affair with Mia	ToM	3	TRUE
Paul believed that Rohan knew that the children were not his	ToM	3	FALSE
In the end, Rohan wondered if Paul had stopped talking to him because he'd felt guilty that Rohan didn't know about the affair	ToM	4	TRUE
Rohan realised that Paul had felt guilty because Rohan didn't know Paul was Anne's father	ToM	4	FALSE
Mia thought Paul knew that she was in love with him, but that he didn't feel the same way about her	ToM	5	TRUE
Paul thought Mia knew that he was in love with her, but that she didn't want to be with him	ToM	5	FALSE
Anne thought it best that Rohan not discover the results of her paternity test because she didn't want him to know that Mia had deceived him	ToM	6	TRUE
Anne thought it best that Rohan not know the results of her paternity test because she didn't want him to know that Mia had never loved him	ToM	6	FALSE
Mia could not afford new earrings	factual	2	TRUE
Mia could not afford a new jumper	factual	2	FALSE
Paul and Rohan were friends from high school where they also met Mia	factual	3	TRUE
Paul and Rohan were friends from work where they also met Mia	factual	3	FALSE
Mia said she was working late when really she was having an affair with Paul	factual	4	TRUE
Mia said she was shopping when really she was having an affair with Paul	factual	4	FALSE

Mia was out shopping for a new jumper when she was the victim of a hit and run that took her life	factual	5	TRUE
Mia was out shopping for a new pair of earrings when she was the victim of a hit and run that took her life	factual	5	FALSE
Rohan, who had been friends with Paul since high school, raised two children with Mia despite their rocky relationship	factual	6	TRUE
Rohan, who had been friends with Paul since high school, raised two children with Mia with whom he had a stable relationship	factual	6	FALSE

Story 3: 'A white lie'

Benji and Nico were planning a romantic weekend away for their anniversary and they both knew it would be a perfect opportunity to propose. Benji wanted to be the one to pop the question, but he hadn't told Nico because he wanted it to be a surprise. Nico thought Benji might be waiting for a proposal from him and it was making him paranoid. Benji's sister Sophie worked at a jewellery shop. She and Benji were close, and Benji often confided in her about his romantic life. Nico was sure she knew whether Benji was waiting for a proposal or not. Nico had noticed that Benji was flustered about the weekend away, and Sophie was being extra smiley around them. Then he saw a bag from Sophie's jewellery shop in the kitchen. Unsure if Benji got something for himself, Nico was just about to peek inside the bag when Benji and Sophie walked in. Sophie laughed and said "beat you to it, Nico, I bought Benji that watch he's been wanting for his birthday." Benji looked relieved.

**Statement**	**Type**	**Order/ Level**	**Expected response**
Benji wanted his proposal to be a surprise	ToM	2	TRUE
Benji wanted Nico to propose	ToM	2	FALSE
Nico wondered whether Benji wanted him to propose	ToM	3	TRUE
Nico knew Benji wanted him to propose	ToM	3	FALSE
Nico thought Sophie knew whether Benji wanted Nico to propose	ToM	4	TRUE
Nico thought Sophie believed that Nico intended to propose to her brother, Benji	ToM	4	FALSE
Sophie knew Benji didn't want Nico to suspect that Benji intended to propose	ToM	5	TRUE
Sophie thought Benji wanted Nico to know that Benji was disappointed Nico didn't buy him a watch	ToM	5	FALSE
Benji was worried that Nico realised that he intended to propose, hence he felt relieved when Sophie tried to throw Nico off the scent	ToM	6	TRUE
Benji was worried that Nico also intended to buy him the watch he wanted from Sophie's shop, hence he felt relieved when Sophie tried to stop him	ToM	6	FALSE
Sophie and Benji are close	factual	2	TRUE
Sophie and Benji have a distant relationship	factual	2	FALSE
Sophie and Benji walked in on Nico when he was about to peek into the bag	factual	3	TRUE
Sophie and Benji walked in on Nico opening a jewellery box	factual	3	FALSE

Benji and Nico were going on a romantic weekend away together for their anniversary	factual	4	TRUE
Benji and Sophie were going on a weekend away together for Sophie's birthday	factual	4	FALSE
Nico saw a bag from the jewellery shop where Sophie worked in the kitchen and nearly peeked inside	factual	5	TRUE
Nico bought a gift from Sophie's shop and Benji, Nico's partner, saw the box in the kitchen	factual	5	FALSE
Sophie told Nico, Benji's partner, that she had bought a gift for Benji for his birthday	factual	6	TRUE
Sophie told Nico, Benji's partner, that she had bought a weekend away for him and Benji to celebrate their engagement	factual	6	FALSE

Story 4: 'False belief'

It was Anna's first night in her beautiful new Victorian flat, and she'd offered to cook her housemates dinner. Arjun and Ben were close friends who'd lived in the flat for years, and while Ben was at the office, Anna asked Arjun if roast chicken would be a good choice for dinner. Arjun, preoccupied with work, waved her off saying "sounds great." When Ben arrived home that night, Anna greeted him at the door and told him what they were going to eat. Ben looked uncomfortable, and said "I'm vegetarian... but I'll be fine eating the side dishes." Anna ran to the kitchen, upset, and asked Arjun why he'd led her to believe that Ben would be happy with chicken. Arjun saw the confusion on her face, apologised and tried to reassure her that it was an honest mistake. Then he bolted out the door to buy some tofu for Ben's dinner. While Arjun was at the shops, Ben explained to Anna that Arjun was dealing with a crisis at work and hadn't been himself lately. Anna felt better.

Statement	Type	Order/ Level	Expected response
Anna thought that Ben would eat chicken	ToM	2	TRUE
Anna knew that Ben was a vegetarian	ToM	2	FALSE
Ben wanted Anna to feel better	ToM	3	TRUE
Ben wanted Anna to feel guilty for cooking the chicken	ToM	3	FALSE
Arjun was worried that Anna thought he'd intentionally lied to her	ToM	4	TRUE
Arjun was worried that Anna thought he was angry with her	ToM	4	FALSE
Ben wanted Anna to know that Arjun would not have intended to mislead her	ToM	5	TRUE
Ben wanted Anna to know that Arjun had believed Ben would like chicken	ToM	5	FALSE
Anna wondered if Arjun knew that Ben wouldn't want chicken and had intended to mislead her about it	ToM	6	TRUE
Anna thought that Arjun knew that Ben would think she was trying to make him feel disregarded by cooking chicken	ToM	6	FALSE
Anna just moved into a new flat	factual	2	TRUE
Anna just moved into a new suburb	factual	2	FALSE

When Ben got home, Anna greeted him at the door	factual	3	TRUE
When Ben got home, Arjun greeted him at the door	factual	3	FALSE
Ben, who is a vegetarian, told Anna he would eat the side dishes	factual	4	TRUE
Ben, who is a vegetarian, told Anna that he would eat the tofu	factual	4	FALSE
After Ben got home, Arjun went to the shop to buy some tofu for Ben because he couldn't have the chicken	factual	5	TRUE
After Ben got home, Arjun went to the shop to buy some tofu for Ben because he complained about the chicken	factual	5	FALSE
Ben, who worked from the office and was a vegetarian, had been living with Arjun for years and become close friends with him	factual	6	TRUE
Ben, who worked from home and liked chicken, had been living with Arjun for years and become close friends with him	factual	6	FALSE

Story 5: 'Patty's intent'

When Sarah uncovered the dusty portfolio of drawings, she knew she'd found a treasure. Sarah was visiting her Grandma Gertrude at the family home in Edinburgh. Gertrude was trying to clear out the house, so when Sarah emerged from the basement with a portfolio of drawings Gertrude had made many years ago in university, she was delighted that Sarah wanted it. The two sat in the kitchen and called Sarah's husband. "Can you order some small frames? Grandma's drawings will make perfect surprise Christmas gifts for the family!" As they got off the phone, Sarah's sister Patty arrived, and Sarah hid the portfolio in her bag. Patty gave Sarah and Gertrude a hug and asked loudly "Did you find anything worth keeping in this mess? How about some of that beautiful art you used to make, grandma? I'd love a second piece on my wall" While Gertrude wasn't looking Sarah shot Patty a look and raised her eyebrows. Last year, Patty had boasted to Sarah about the great price she got for the painting Grandma Gertrude had made her for her birthday. Sarah left in a hurry, with her purse closed tightly under her arm. "See you at Christmas!" Sarah said.

**Statement**	**Type**	**Order/ Level**	**Expected response**
Sarah wanted to give her family members framed drawings for Christmas	ToM	2	TRUE
Sarah wanted to sell the drawings she found in the basement	ToM	2	FALSE
Sarah suspected that Patty wanted to sell Gertrude's drawings	ToM	3	TRUE
Sarah was worried that Gertrude didn't want her to take the drawings	ToM	3	FALSE
Gertrude knew that Sarah didn't want Patty to know about the drawings	ToM	4	TRUE
Gertrude knew that Sarah thought Patty wanted to sell the drawings	ToM	4	FALSE
Sarah wanted Patty to know that she suspected Patty was lying about having the painting Grandma Gertrude made her on her wall	ToM	5	TRUE
Sarah wanted Patty to know that she suspected Gertrude was hoping to sell the drawings	ToM	5	FALSE
Sarah suspected Patty wanted Gertrude to believe that she would like to put Gertrude's art up on her walls so that Gertrude would feel more inclined to give it to	ToM	6	TRUE

her			
Sarah knew Patty wanted Gertrude to think that she would like to help Gertrude sell her art so that Gertrude would feel more inclined to give it to her	ToM	6	FALSE
The portfolio of drawings was found in Grandma Gertrude's basement	factual	2	TRUE
Patty found the portfolio of drawings	factual	2	FALSE
Patty arrived at Gertrude's house after Sarah	factual	3	TRUE
Patty saw the portfolio in Sarah's bag before Sarah left	factual	3	FALSE
Sarah was with Gertrude in the kitchen when she called her husband	factual	4	TRUE
Gertrude's granddaughter, Patty, talked to Sarah's husband on the phone	factual	4	FALSE
Gertrude created the portfolio of drawings that Sarah found in the basement while she was in university	factual	5	TRUE
Sarah created the portfolio of drawings that Grandma Gertrude found in the basement when she and Patty were children	factual	5	FALSE
Sarah was visiting her Grandma, Gertrude, in Edinburgh when she found the portfolio of drawings in the basement	factual	6	TRUE
Sarah was visiting her sister, Patty, in Edinburgh when she found the portfolio of drawings that Grandma Gertrude had made	factual	6	FALSE

Story 6: 'Beverly's worry'

All summer, Ryland's favourite activity was playing football in the garden with his dad Steven. In the autumn, Ryland's mother, Beverly, signed Ryland up for a football club at the local community centre. Ryland had been getting bullied at school recently so Beverly was worried that he would find the club intimidating, and thought that going with a friend would help. Beverly suggested to Steven that they find out if Luna, one of Ryland's classmates, could join the club. Beverly and Steven called Luna's mum, Angie, and Beverly asked, "Do you think Luna would want to join Ryland's football club? I heard her talking about it on the playground!" Steven pats Beverly's arm and whispers "Is that true?! Luna hates sport, and Angie knows it!" Meanwhile, Angie considers. Luna doesn't like team sports, so she wouldn't have mentioned football at school, but Angie knew that Ryland was being bullied so thought that Beverly was inventing an excuse to get Luna to join. "Sure," said Angie, "maybe Luna will see that sport can be fun."

**Statement**	**Type**	**Order/ Level**	**Expected response**
Ryland enjoys playing football with his dad	ToM	2	TRUE
Luna enjoys playing football	ToM	2	FALSE
Angie suspected that Beverly was lying about what Luna said	ToM	3	TRUE
Angie suspected that Beverly was lying about signing Ryland up to football club	ToM	3	FALSE
Beverly hoped Angie would believe that Luna wanted to play football	ToM	4	TRUE
Steven thought that Angie would believe that Luna wanted to play football	ToM	4	FALSE
Steven was concerned that Angie would know Beverly was lying about Luna wanting	ToM	5	TRUE

to play football			
Steven was concerned that Angie would believe Beverly knew that Luna wanted to play football	ToM	5	FALSE
Angie suspected that Beverly thought Angie would want to sign Luna up for football club if she believed that Luna wanted to play	ToM	6	TRUE
Angie suspected that Beverly thought Angie would not sign Luna up for football club if she knew that Ryland wouldn't want to be there without Luna	ToM	6	FALSE
Football club is at the local community centre	factual	2	TRUE
Football club is in the next door town	factual	2	FALSE
Beverly called Angie to talk about football club	factual	3	TRUE
Angie called Beverly to talk about Ryland being bullied	factual	3	FALSE
Beverly suggested that they call Angie about football club in the autumn	factual	4	TRUE
Steven played football in the garden with Ryland and Luna over the summer	factual	4	FALSE
Beverly signed Ryland, her son, up for a club at the community centre in the autumn	factual	5	TRUE
Beverly signed Luna, Angie's daughter, up for a club at the community centre with Ryland	factual	5	FALSE
Ryland, who spent all summer playing football with his Dad, Steven, is friends with Angie's daughter, Luna	factual	6	TRUE
Ryland, who spent all summer playing football with his friend, Luna, is signed up to football with his dad, Steven	factual	6	FALSE

Story 7: 'Tamara's nerves'

"I've never performed for an audience before", Tamara said to her boyfriend, John, over coffee. "I'm terrified I'm going to mess up Kelly's wedding with a bad performance." John grabbed Tamara's arm and said "don't worry, Kelly just wants you to be part of it." "Yeah, I guess. I better do some extra practice, though", said Tamara. A week later, as Kelly walked down the aisle, Tamara sang 'Here There Everywhere' by The Beatles. Kelly noticed Tamara's hands were shaking, so when she caught her eye, mouthed at her "you got this!" and Tamara began to relax. At the reception, Kelly found Tamara and John and gave Tamara a tight hug. "That was beautiful!" Kelly said, "I didn't want to tell you before because I knew you were already nervous, but that was the last song I listened to with my father before he passed! It means a lot to me." Tamara's eyes grew wide. "Wow...well I'm honoured to have played it." When Kelly left Tamara turned to John and said "Phew, that was a wise move from Kelly! I'm not sure I'd have managed that if I'd known what that song meant to her."

Statement	Type	Order/ Level	Expected response
Tamara felt nervous about performing at Kelly's wedding	ToM	2	TRUE
Tamara felt relaxed about performing at Kelly's wedding	ToM	2	FALSE
Tamara wanted to make Kelly happy by performing at her wedding	ToM	3	TRUE
Tamara wanted to make John jealous by performing at Kelly's wedding	ToM	3	FALSE

John wanted to reassure Tamara that Kelly wouldn't be upset if the performance wasn't perfect	ToM	4	TRUE
John wanted Tamara to know that Kelly would be upset if the performance wasn't perfect	ToM	4	FALSE
Kelly suspected Tamara would be more nervous about performing if Tamara knew that Kelly wanted her to play the song as a tribute to her dad	ToM	5	TRUE
Kelly thought Tamara would have wanted to play a different song if she had known that Kelly wanted her to play the Beatles as a tribute to her dad	ToM	5	FALSE
Tamara was relieved that Kelly realised Tamara would have been more nervous about the performance if she'd known how Kelly felt about the song	ToM	6	TRUE
Tamara felt embarrassed that Kelly knew Tamara would have been more nervous if she'd known how Kelly felt about the song	ToM	6	FALSE
Tamara performed at Kelly's wedding	factual	2	TRUE
Tamara performed at Kelly’s work event	factual	2	FALSE
Kelly's wedding was Tamara's first performance to an audience	factual	3	TRUE
Kelly's wedding was Tamara's third performance to an audience	factual	3	FALSE
Tamara sang a song by The Beatles at Kelly's wedding as Kelly walked down the aisle	factual	4	TRUE
Tamara played a song by The Beatles on the flute at Kelly's wedding	factual	4	FALSE
Kelly hugged Tamara at the wedding reception after Tamara had performed at Kelly's wedding	factual	5	TRUE
Kelly hugged John at the wedding reception after Tamara had performed at Kelly's wedding	factual	5	FALSE
Kelly chose 'Here There Everywhere' for her wedding because it was the last song she'd listened to with her father before he passed	factual	6	TRUE
Kelly chose 'Here There Everywhere' for her wedding because it was the last song she'd listened to with her mother before she passed	factual	6	FALSE
